# Silver triflate catalyzed synthesis of 3-aminoalkylated indoles and evaluation of their antibacterial activities

**DOI:** 10.1186/2191-2858-1-10

**Published:** 2011-09-27

**Authors:** Vagicherla Kameshwara Rao, Madharam Sudershan Rao, Navin Jain, Jitendra Panwar, Anil Kumar

**Affiliations:** 1 Department of Chemistry, Birla Institute of Technology and Science, Pilani 333 031, Rajasthan, India; 2 Department of Biological Sciences, Birla Institute of Technology and Science, Pilani 333 031, Rajasthan, India

**Keywords:** 3-Substituted indole, one-pot synthesis, silver triflate, antibacterial agents, multicomponent reactions

## Abstract

An efficient, one-pot synthesis was developed for 3-aminoalkylated indoles by three-component coupling reaction of aldehydes, *N*-methylanilines, and indoles using AgOTf as a catalyst. A series of twenty 3-aminoalkylated indoles was evaluated for their antibacterial activities against both Gram negative and Gram positive bacteria. Compounds **4b **and **4r **showed good antibacterial activity against both Gram positive and Gram negative strains. However, inversing the property of substituent (from **4r **to **4q**) resulted in the significant fall in the magnitude of antibacterial activity against *Escherichia coli*.

## Introduction

Antimicrobial resistance continues to grow quickly among key microbial pathogens and has become a severe global problem in recent years. Bacterial resistance to almost all available antibacterial agents has been reported [1]. Because of this many infectious diseases, such as HIV infection, staphylococcal infection, tuberculosis, influenza, gonorrhea, candida infection, and malaria, are becoming difficult to treat. Thus, along with trying to control bacterial resistance there is an urgent need for new potent classes of antibiotics with novel modes of action.

**Figure 1:**
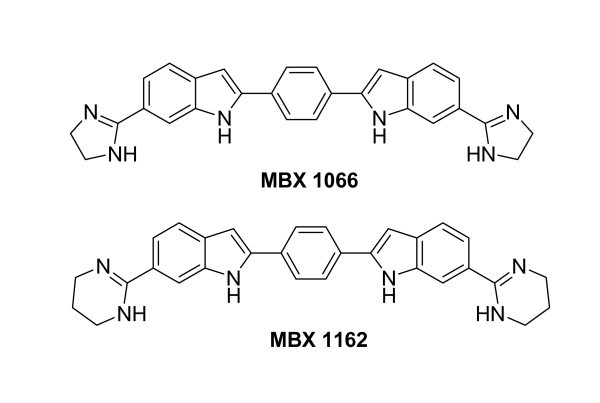
**Chemical structure of *bis*-indole derivatives used as antibacterial agents**.

The indole scaffold is a prominent and privileged structural motif which is embodied in a myriad of natural products and molecules of pharmaceutical interest in a variety of therapeutic areas [2,3]. They possess a wide spectrum of biological activities such as antibacterial [4], anticonvulsant, and antihypertensive activity. *bis*-Indole-based compounds have been reported to have broad-spectrum antibacterial activities against antibiotic-resistant strains and are currently being pursued as topical agents (Figure 1) [5-7]. 1,2,3,4-Tetrahydropyrazino [1,2] indoles [8] and triazino [[8],6-b] indoles [9] have been reported to have antifungal properties. Hapalindole A isolated from the blue green algae *Hapalosiphon fontinalis *is a 3-substituted indole derivative. It exhibits potent antibacterial and antimycotic activities [10]. The antibacterial activity of 3-substituted indole derivatives has not been much studied. Owing to interesting chemical and biological properties of indole molecules, development of efficient methods that allow rapid access to functionalized indoles with different substitution patterns constitutes an emerging area in organic synthesis.

Multicomponent reactions have received a great attention of organic chemists as they can provide drug-like molecules with several degrees of structural diversity in a one-pot operation and offer significant advantages over conventional linear-type syntheses such as high atom economy and E-factors, low cost, reduction in overall reaction time, and operational simplicity. There are only a few methods available for the synthesis of 3-aminoalkylated indoles which have been found in many natural products. Recently, a one-pot multicomponent method was developed for the synthesis of 3-aminoalkylated indoles by the reaction of aldehyde, amine, and indole [11-14]. The reaction requires longer time, high temperature, and is generally accompanied by formation of *bis*-indolyl compound. Thus, there is still high need for the development of an efficient and straightforward method for the synthesis of 3-substituted indole derivatives. In continuation to our interest in novel reaction methodologies under environmentally friendly conditions [15,16], we herein report an efficient silver triflate catalyzed synthesis of 3-aminoalkylated indoles and their antibacterial activities.

## Experimental

### General

Melting points were determined in open capillary tubes on a MPA120-Automated Melting Point apparatus and are uncorrected. The^1^H and^13^C NMR spectra were recorded on a Bruker Heaven 11400 (400 MHz) spectrometer using TMS as internal standard and the chemical shifts are expressed in ppm. All the metal triflates, indole, *N*-methylaniline, and aldehydes were purchased from Sigma-Aldrich. The products were purified by column chromatography using silica gel (60-120 mesh, S. D. Fine, India). The bacterial cultures (*Bacillus subtilis *MTCC 121, *Staphylococcus aureus *MTCC 96 and *Escherichia coli *MTCC 1652) were procured from Microbial Type Culture Collection, Institute of Microbial Technology, Chandigarh, India. Dimethyl sulfoxide (DMSO) and chloramphenicol (standard broad spectrum antibiotic) were used as negative and positive controls, respectively. The experiments were carried out in triplicates.

### General procedure for preparation of 3-aminoalkylated indoles (4)

To a solution of *N*-methylaniline (114 mg, 1.2 mmol) and a benzaldehyde (1.0 mmol) in acetonitrile (10 mL), AgOTf (30 mg) was added. The reaction mixture was stirred at room temperature. After 30 min, indole or *N*-methylindole (0.71 mmol) was added to the reaction and the mixture was allowed it to stir for an additional 90 min. The progress of reaction was monitored by TLC. After completion of the reaction, solvent was removed under reduced pressure. To the residue, diethyl ether was added and filtered. The filtrate was dried over anhydrous sodium sulfate and concentrated to obtain the crude product, which was purified by column chromatography on silica gel (100-200 mesh) using ethyl acetate/hexane as eluents to yield a pure product (**4a-4r**). All the compounds were characterized by ESI-MS,^1^H NMR, and^13^C NMR spectroscopic data.

### *N*-((4-Chlorophenyl)(1H-indol-3-yl)methyl)-*N*-methylbenzenamine (4a)

Brown solid, m.p. 183-185°C;^1^H NMR (400 MHz, CDCl_3_): δ 7.98 (s, 1H), 7.37 (d, *J *= 4.0 Hz, 2H), 7.27-7.16 (m 5H), 7.03 (d, *J *= 8.0 Hz, 3H), 6.57 (d, *J *= 8.0 Hz, 3H), 5.55 (s, 1H), 2.83 (s, 3H);^13^C NMR (100 MHz, DMSO-*d*_6_): δ 147.5, 141.6, 137.0, 135.5, 132.9, 129.8, 129.1, 129.0, 127.3, 124.2, 121.9, 120.8, 120.2, 119.3, 113.1, 111.2, 47.6, 31.0, 21.1; ESI-MS (*m/z*): 346.9975 [M + H]^+^.

### *N*-((1H-Indol-3-yl)(p-tolyl)methyl)-*N*-methylbenzenamine (4b)

Brown solid, m.p. 136-138°C;^1^H NMR (400 MHz, DMSO-*d*_6_): δ 10.62 (s, 1H), 7.31 (d, *J *= 8.0 Hz 1H), 7.07-6.98 (m, 7H), 6.92-6.90 (m, 1H), 6.62-6.61 (m, 1H), 6.43 (d, *J *= 8.4 Hz, 2H), 5.46 (s, 1H), 5.40 (s, 1H), 2.49-2.48 (m, 3H), 2.24 (s, 1H);^13^C NMR (100 MHz, DMSO-*d*_6_): δ 147.6, 141.7, 136.7, 135.4, 133.2, 129.7, 128.9, 128.8, 127.2, 123.9, 121.9, 120.9, 120.1, 119.3, 112.4, 111.0, 47.6, 31.0, 21.1; ESI-MS (*m/z*): 327.0665 [M + H]^+^.

### *N*-((1H-Indol-3-yl)(4-methoxyphenyl)methyl)-*N*-methylbenzenamine (4c)

Brown solid, m.p. 177-179°C;^1^H NMR (400 MHz, CDCl_3_): δ 7.93 (s, 1H), 7.42-7. 26 (m, 3H), 7.16 (d, *J *= 7.6 Hz, 3H), 7.06-6.98 (m, 3H), 6.83 (d, *J *= 7.6, 2H), 6.56 (d, *J *= 8.0 Hz, 3H), 5.54 (s, 1H), 3.80 (s, 3H), 2.83 (s, 3H);^13^C NMR (100 MHz, CDCl_3_): δ 168.98, 157.90, 147.56, 136.99, 133.29, 129.85, 129.65, 127.13, 123.90, 121.97, 120.16, 119.27, 113.56, 112.40, 110.97, 55.21, 47.13, 30.95; ESI-MS (*m/z*): 343.0446 [M + H]^+^.

### *N*-((1H-Indol-3-yl)(phenyl)methyl)-*N*-methylbenzenamine (4d)

Brown solid, mp 189-191°C;^1^H NMR (400 MHz, DMSO-*d*_6_): δ 10.01 (s, 1H), 7.57 (s, 1H), 7.35 (d, *J *= 8.0 Hz, 1H), 7.26-7.20 (m, 4H), 7.17-7.14 (m, 2H), 7.09-6.86 (m, 3H), 6.60 (d, *J *= 2.0 Hz, 3H), 5.53 (s, 1H), 2.79 (s, 3H);^13^C NMR (100 MHz, CDCl_3_): δ 147.7, 144.9, 133.0, 130.1, 129.8, 129.0, 128.2, 127.2, 124.0, 122.0, 120.7, 120.1, 119.3, 112.4, 111.0, 48.0, 31.0; ESI-MS (*m/z*): 313.0450 [M + H]^+^.

### *N*-((1H-Indol-3-yl)(4-hydroxy phenyl)methyl)-*N*-methylbenzenamine (4e)

Brown solid, m.p. 139-140°C;^1^H NMR (400 MHz, CDCl_3_): δ 7.929 (s, 3H), 7.41-7.01 (m, 7H), 6.75-6.57 (m, 4H), 5.84 (s, 1H), 5.31 (s, 1H), 2.83 (s, 3H);^13^C NMR (100 MHz, CDCl_3_): δ 153.8, 136.8, 133.5, 130.5, 130.1, 129.9, 127.1, 123.9, 123.6, 122.0, 120.0, 119.3, 115.1, 112.3, 111.1, 47.1, 39.5, 31.0; ESI-MS (*m/z*): 329.0347 [M + H]^+^.

### *N*-((4-Hydroxy-3-bromophenyl)(1H-indol-3-yl)methyl)-*N*-methylbenzenamine (4f)

Brown solid, m.p. 187-189°C;^1^H NMR (400 MHz, CDCl_3_): δ 7.97 (s, 1H), 7.38-7.19 (m, 4H), 7.08-6.93 (m, 5H), 6.59 (s, 3H), 5.50 (s, 1H), 2.84 (s, 3H);^13^C NMR (100 MHz, CDCl_3_): δ 150.3, 147.6, 138.5, 136.9, 132.0, 129.7, 129.6, 127.0, 123.9, 123.6, 122.6, 122.1, 119.9, 119.4, 115.7, 112.6, 111.1, 110.1, 46.9, 31.0; ESI-MS (*m/z*): 406.9101 [M + H]^+^.

### *N*-((1H-Indol-3-yl)(3-methoxyphenyl)methyl)-*N*-methylbenzenamine (4g)

Brown solid, m.p. 136-139°C;^1^H NMR (400 MHz, CDCl_3_): δ 7.939 (s, 2H), 7.43-7.22 (m, 3H), 7.20-7.17 (m, 3H), 7.08-6.86 (m, 2H), 6.84-6.81 (m, 2H), 6.57 (d, *J *= 7.6 Hz, 2H), 5.56 (s, 1H), 3.75 (s, 3H), 2.83 (s, 3H);^13^C NMR (100 MHz, CDCl_3_): δ 159.5, 148.5, 147.6, 146.5, 145.8, 136.7, 132.9, 129.7, 127.1, 123.9, 123.6, 122.0, 121.6, 120.1, 120.0, 115.0, 112.5, 111.2, 110.1, 55.5, 48.0, 31.0; ESI-MS (*m/z*): 343.0968 [M + H]^+^.

### *N*-((1H-Indol-3-yl)(2,4-dimethoxyphenyl)methyl)-*N*-methylbenzenaminen (4h)

Brown solid, m.p. 123-126°C;^1^H NMR (400 MHz, CDCl_3_): δ 7.91 (s, 1H), 7.34-7.27 (m, 2H), 7.16-6.92 (m, 4H), 6.57-6.37 (m, 4H), 5.59 (s, 1H), 3.79 (s, 6H), 2.83 (s, 3H);^13^C NMR (100 MHz, CDCl_3_): δ 159.9, 157.9, 147.3, 136.8, 130.2, 130.0, 129.7, 125.7, 125.0, 123.9, 121.8, 120.2, 119.1, 112.4, 112.3, 110.9, 103.8, 95.6, 55.7, 55.3, 39.1, 31.1; ESI-MS (*m/z*): 373.0427 [M + H]^+^.

### *N*-Methyl-*N*-((1-methyl-1H-indol-3-yl)(phenyl)methyl)benzenamine (4i)

Brown solid, mp 189-191°C;^1^H NMR (400 MHz, CDCl_3_): δ 7.28-7.22 (m, 8H), 7.04 (d, *J *= 8.0 Hz, 4H), 6.58-6.45 (m, 3H), 5.59 (s, 1H), 3.71 (s, 3H), 2.84 (s,3H);^13^C NMR (100 MHz, CDCl_3_): δ 129.73, 128.97, 128.71, 128.35, 128.18, 125.94, 124.55, 122.32, 121.67, 121.52, 121.52, 120.18, 119.04, 118.72, 112.37, 109.06, 94.27, 47.90, 32.68, 30.93. ESI-MS (*m/z*): 327.055 [M + H]^+^.

### *N*-((4-Chlorophenyl)(1-methyl-1H-indol-3-yl)methyl)-*N*-methylbenzenamine (4j)

Brown solid, m.p. 208-210°C;^1^H NMR (400 MHz, CDCl_3_): δ 7.29-7.18 (m, 7H), 7.03 (d, *J *= 8.0 Hz, 3H), 6.57 (d, *J *= 4.0 Hz, 4H), 6.42 (s, 1H), 5.54 (s, 1H), 3.71 (s, 3H), 2.84 (s, 3H);^13^C NMR (100 MHz, CDCl_3_): δ 147.8, 143.5, 137.5, 132.5, 131.6, 130.9, 130.3, 130.2, 129.7, 129. 6, 128.7, 128.5, 128.3, 121.7, 120.5, 118.9, 118.6, 112.6, 112.4, 109.2, 47.3, 32.7, 30.9. ESI-MS (*m/z*): 361.002 [M + H]^+^.

### *N*-Methyl-*N*-((1-methyl-1H-indol-3-yl)(p-tolyl)methyl)benzenamine (4k)

Brown solid, m.p. 202-204°C;^1^H NMR (400 MHz, CDCl_3_): δ 7.31-7.26 (m, 3H), 7.19-7.01 (m, 8H), 6.57 (d, *J *= 8.0 Hz, 2H), 6.46 (s, 1H), 5.56 (s, 1H), 3.71 (s, 3H), 2.84 (s, 3H), 2.35 (s, 3H);^13^C NMR (100 MHz, CDCl_3_): δ 147.6, 142.0, 137.5, 135.3, 133.9, 129.7, 128.9, 128.8, 128.7, 127.5, 121.5, 120.2,119.2, 118.7, 112.4, 109.0, 47.5, 32.7, 31.0, 21.1; ESI-MS (*m/z*): 341.066 [M + H]^+^.

### *N*-((1H-indol-3-yl)(3-nitrophenyl)methyl)-*N*-methylbenzenamine (4l)

Brown solid, m.p. 193-194°C;^1^H NMR (400 MHz, CDCl_3_): δ 8.13-8.07 (m, 3H), 7.59 (d, *J *= 8.0 Hz, 1H), 7.46-7.38 (m, 2H), 7.21 (d, *J *= 8.0 Hz, 2H), 7.06-7.02 (m, 3H), 6.66-6.57 (m, 3H), 5.68 (s, 1H), 2.84 (s, 3H);^13^C (100 MHz, CDCl_3_): δ 148.1, 147.8, 146.8, 136.5, 134.9, 130.9, 129.4, 128.8, 126.4, 123.8, 123.5, 122.1, 121.1, 119.4, 119.4, 119.1, 112.3, 112.2, 111.0, 47.5, 30.6; ESI-MS (*m/z*): 358.007 [M + H]^+^.

### *N*-((5-Bromo-1H-indol-3-yl)(phenyl)methyl)-*N*-methylbenzenamine (4m)

Brown solid, m.p. 207-209°C;^1^H NMR (400 MHz, CDCl_3_): δ 8.07 (s, 1H), 7.39 (s, 1H), 7.27-7.23 (m, 7H), 7.02 (d, *J *= 8.0 Hz, 2H), 6.58 (d, *J *= 8.0 Hz, 3H), 5.52 (s, 1H), 2.83 (s, 3H);^13^C NMR (100 MHz, CDCl_3_): δ 147.5, 144.3, 135.4, 132.7, 129.7, 128.9, 128.3, 126.2, 125.2, 125.0, 122.5, 120.4, 112.7, 112.5, 47.7, 31.1; ESI-MS (*m/z*): 390.9823 [M + H]^+ ^and 392.9105 [M + 2 + H]^+^.

### *N*-((5-Methoxy-1H-indol-3-yl)(4-methoxyphenyl)methyl)-*N*-methylbenzenamine (4n)

Brown solid, m.p. 203-205°C;^1^H NMR (400 MHz, DMSO-*d*_6_): δ 10.62 (s, 1H), 7.24-7.20 (m, 3H), 7.09 (d, *J *= 4.0 Hz, 1H), 6.91 (d, *J *= 8.0 Hz, 1H), 6.81 (d, *J *= 8.0 Hz, 2H), 6.77 (s, 1H), 6.70-6.66 (m, 3H), 6.60-6.53 (m, 1H), 6.43 (d, *J *= 8.0 Hz, 1H), 5.66 (s, 1H), 3.68 (s, 3H), 3.44 (s, 3H), 2.48 (s, 3H);^13^C NMR (100 MHz, DMSO-*d*_6_): δ 157.8, 153.1, 137.7, 132.3, 129.9, 129.7, 129.4, 127.4, 124.7, 118.5, 113.8, 112.5, 111.9, 111.0, 102.0, 55.7, 55.4, 47.0, 30.4; ESI-MS (*m/z*): 373.1681 [M + H]^+^.

### *N*-((5-Methoxy-1H-indol-3-yl)(*p*-tolyl)methyl)-*N*-methylbenzenamine (4o)

Brown solid m.p. 197-199°C;^1^H NMR (400 MHz, DMSO-*d*_6_): δ 10.60 (s, 1H), 7.21 (d, *J *= 4.0 Hz, 3H), 7.06 (d, *J *= 8.0 Hz, 3H), 6.91 (d, *J *= 4.0 Hz, 1H), 6.77 (s, 1H), 6.69-6.66 (m, 3H), 6.59-6.52 (m, 1H), 6.42 (d, *J *= 8.0 Hz, 1H), 5.66 (s, 1H), 3.57 (s, 3H), 2.48 (s, 3H), 2.23 (s, 3H);^13^C NMR (100 MHz, DMSO-*d*_6_): δ 153.1, 148.9, 142.5, 135.0, 132.3, 129.5, 129.1, 128.9, 128.7, 127.5, 124.7, 118.3, 112.4, 111.9, 110.9, 102.0, 55.7, 47.4, 30.4, 21.1; ESI-MS (*m/z*): 357.1747 [M + H]^+^.

### *N*-((5-Bromo-1H-indol-3-yl)(*p*-tolyl)methyl)-*N*-methylbenzenamine (4p)

Brown solid, m.p. 195-197°C;^1^H NMR (400 MHz, DMSO-*d*_6_): δ 11.02 (s, 1H), 7.29 (d, *J *= 8.0 Hz, 1H), 7.19 (d, *J *= 4.0 Hz, 1H), 7.11 (d, *J *= 4.0 Hz, 1H), 7.08-7.00 (m, 4H), 6.89 (d, *J *= 4.0 Hz, 2H), 6.75 (d, *J *= 8.0 Hz, 1H), 6.68 (d, *J *= 4.0 Hz, 1H), 6.42 (d, *J *= 8.0 Hz, 2H), 5.39 (s, 1H), 2.48 (s, 3H), 2.22 (s, 3H);^13^C NMR (100 MHz, DMSO-*d*_6_): δ 148.7, 142.4, 135.7, 135.3, 131.7, 129.4, 129.2, 128.7, 126.0, 123.9, 121.7, 119.4, 114.0, 112.0, 111.3, 47.0, 30.4, 21.1; ESI-MS (*m/z*): 405.0683 [M + H]^+ ^and 407.072 [M + 2 + H]^+^.

### 4-Chloro-*N*-((4-chlorophenyl)(5-methoxy-1H-indol-3-yl)methyl)benzenamine (4q)

Brown solid, m.p. 190-191°C;^1^H NMR (400 MHz, DMSO-*d*_6_): δ 11.02 (s,1H), 7.29 (d, *J *= 4.0 Hz, 1H), 7.19 (s, 1H), 7.11 (d, *J *= 4.0 Hz, 1H), 7.07 (d, *J *= 4.0 Hz, 2H), 6.89 (d, *J *= 4.0 Hz, 2H), 6.83 (d, *J *= 8.0 Hz, 3H), 6.67 (s, 1H), 6.43 (d, *J *= 4.0 Hz, 2H), 5.39 (s, 1H), 3.69 (s, 3H), 2.47(s, 3H);^13^C NMR (100 MHz, DMSO-*d*_6_): δ 157.8, 148.6, 137.4, 135.8, 131.7, 129.8, 129.4, 128.9, 126.0, 123.9, 121.8, 119.3, 114.0, 112.0, 111.1, 55.4, 46.6, 30.4; ESI-MS (*m/z*): 421.0695 [M + H]^+^.

### *N*-((4-Chlorophenyl)(5-methoxy-1H-indol-3-yl)methyl)-*N*-methylbenzenamine (4r)

Brown solid, m.p. 201-203°C;^1^H NMR (400 MHz, DMSO-*d*_6_): δ 10.60 (s, 1H), 7.31-7.28 (m, 5H), 7.22-7.17 (m, 2H), 6.91 (s, 1H), 6.79 (s, 1H), 6.69 (s, 2H), 6.52 (s, 1H), 6.43 (d, *J *= 4.0 Hz, 2H), 5.73 (s, 1H), 3.30 (s, 3H), 2.47(s, 3H);^13^C NMR (100 MHz, DMSO-*d*_6_): δ 153.2, 148.9, 144.6, 132.3, 131.2, 130.8, 130.6, 129.5, 128.4, 127.3, 125.3, 125.2, 124.8, 117.7, 112.6, 112.0, 111.1, 55.7, 47.3, 30.3; ESI-MS (*m/z*): 377.1223 [M + H]^+^.

### *N*-((4-chlorophenyl)(5-methoxy-1H-indol-3-yl)methyl)benzenamine (4s)

Brown solid, m.p. 198-201°C;^1^H NMR (400 MHz, MeOH) δ 7.258-7.164 (m, 6H), 6.95 (d, *J *= 8.0 Hz, 2H), 6.73-6.55 (m, 3H), 6.53 (d, *J *= 10.0 Hz, 2H), 5.47 (s, 1H), 3.61 (s, 3H);^13^C NMR (100 MHz, DMSO-*d*_6_): δ 154.7, 148.3, 141.3, 132.3, 130.9, 129.3, 128.9, 128.7, 128.2, 123.4, 117.5, 112.5, 112.3, 112.1, 110.7, 109.5, 55.3; ESI-MS (*m/z*): 363.21 [M + H]^+^, 365.20 [M + H +2]^+^.

### Anti-bacterial assay

Zone of inhibition assay was performed at 128 μg mL^-1 ^concentration for all the compounds (**4a-s**) using disk diffusion method [17]. For this purpose, Mueller-Hilton (HiMedia, India) agar medium was prepared and sterilized by autoclaving at 121°C at 15 psi for 15 min. The medium was poured into sterile Petri dishes under aseptic conditions using laminar air flow chamber. After the solidification of medium, the suspension of the test organism (10^6 ^cfu mL^-1^) was swabbed onto the individual media plates using a sterile glass spreader. A sterile disk (9-mm diameter) impregnated with compound was placed over media surface and the plates were incubated at 37°C for 18-24 h under dark conditions. The determination as to whether the organism is susceptible, intermediate, or resistant was made by measuring the size of zone of inhibition in comparison with standard antibiotic.

MIC assay was performed to determine the lowest concentration of compound necessary to inhibit a test organism. MIC values were evaluated for all the compounds (**4a-t**) using broth microdilution method as per the standard guidelines [18]. Assay was carried out for the compounds at 0.5, 1.0, 2.0, 4.0, 8.0, 16.0, 32.0, 64.0, 128.0 μg mL^-1 ^concentrations. A set of tubes containing Muller Hilton broth medium with different concentrations of compounds were prepared. The tubes were inoculated with bacterial cultures (10^6 ^cfu mL^-1^) and incubated on a rotary shaker (180 rpm) at 37°C for 18-24 h under dark conditions. MIC values were defined as lowest concentration of compound that prevented the visible growth of bacteria after the incubation period. All the experiments were performed in three replicates.

## Results and discussion

### Chemistry

Initially, we investigated reaction of indole benzaldehyde, and *N*-methylaniline to give **4a **in acetonitrile using different Lewis acid catalysts (Table 1). Among different catalysts studied AgOTf gave highest yield of **4a **(Table 1, entry 2). Among other catalysts, Ce(OTf)_3_, Yb(OTf)_3 _and pTSA gave good yield of **4a **(Table 1, entries 7, 10, and 12). Formation of *bis*(indolyl)methane (5-32%) as side product was observed with most of the catalyst studied except with AgOTf, Ce(OTf)_3_, and Yb(OTf)_3_.

**Table 1:** Optimization of reaction condition for model reaction generating 4a

Number	Catalyst	(Catalyst mol %)	Solvent	Time (h)	Yield (%)^a^
1	AgOTf	1	CH_3_CN	4	58
2	AgOTf	5	CH_3_CN	4	78
3	AgOTf	10	CH_3_CN	4	86
4	Sc(OTf)_3_	10	CH_3_CN	4	43
5	Ga(OTf)_3_	10	CH_3_CN	4	45
6	Zn(OTf)_2_	5	CH_3_CN	4	52
7	Ce(OTf)_3_	5	CH_3_CN	4	71
8	Cu(OTf)_2_	5	CH_3_CN	4	68
9	Ba(OTf)_2_	5	CH_3_CN	4	50
10	Yb(OTf)_3_	5	CH_3_CN	4	72
11	FeCl_3_	5	CH_3_CN	2	59
12	pTSA	5	CH_3_CN	3	71
13	BF_3_.OEt_2_	5	CH_3_CN	5	34
14	Mont. K-10	-^b^	CH_3_CN	6	15
15	SiO_2_	-^b^	CH_3_CN	6	30
16	AgOTf	10	DCM	10	67
17	AgOTf	10	DMSO	10	72
18	AgOTf	10	DMF	10	62
19	AgOTf	10	THF	10	58
20	AgOTf	10	[bmim][BF_4_]	12	31^c^
21	AgOTf	10	H_2_O	12	-^d^

^a^Isolated yield

^b^100 mg mole of benzaldehyde

^c^Imine was formed as major product

^d^No product formation was observed.

Subsequently, we investigated different solvents such as DCM, DMSO, DMF, THF, acetonitrile, and ionic liquid [bmim][BF_4_] for the model reaction. Acetonitrile was found to give highest yield of **4a **among all the screened solvent. In case of ionic liquid [bmim][BF_4_] imine was major product. In other solvents substrate did not consume completely and there was mixture of starting material, imine, and **4a**. For further studies we selected AgOTf (10 mol%) as catalyst and acetonitrile as reaction medium of choice.

After determining the optimized reaction conditions, we next studied the substrate scope by taking indoles, aldehydes, and *N*-methyl anilines bearing different substituent for the synthesis of 3-aminoalkylated indoles (**4**). The results are summarized in Table 2. The structure of the synthesized compounds was confirmed by^1^H NMR,^13^C NMR, and mass spectroscopic data. A wide range of structurally diverse aldehydes gave the corresponding product **4 **in good to excellent yields. Aromatic aldehyde having an electron-donating group gave higher yield as compared to aromatic aldehydes with electron withdrawing group (entry 12, Table 2). The reaction was equally effective for *N*-methylindole and 5-unsubstituted indoles affording the desired 3-aminoalkylated indoles in almost equally high yields (entries 13-18, Table 2). However, poor yield of corresponding 3-substituted indole was obtained from aniline (entry 19, Table 1). When aliphatic amines were used it did not result in 3-substituted indole under these conditions.

**Table 2:** Synthesis of 3-aminoalkylated indoles (4a-t) catalyzed by AgOTf

Number	R	R'	R''	R'''	Product	Yield (%)^a^
1	H	H	4-Cl	CH_3_	**4a**	86^b^
2	H	H	4-CH_3_	CH_3_	**4b**	85
3	H	H	4-CH_3_O	CH_3_	**4c**	84
4	H	H	H	CH_3_	**4d**	76
5	H	H	4-OH	CH_3_	**4e**	77
6	H	H	3-Br, 4-OH	CH_3_	**4f**	85
7	H	H	3-CH_3_O	CH_3_	**4g**	83
8	H	H	2,4-CH_3_O	CH_3_	**4h**	80
9	H	CH_3_	H	CH_3_	**4i**	77
10	H	CH_3_	4-Cl	CH_3_	**4j**	76
11	H	CH_3_	4-CH_3_	CH_3_	**4k**	77
12	H	H	3-NO_2_	CH_3_	**4l**	48
13	5-Br	H	H	CH_3_	**4m**	75
14	5-OCH_3_	H	4-OCH_3_	CH_3_	**4n**	85
15	5-OCH_3_	H	4-CH_3_	CH_3_	**4o**	82
16	5-Br	H	4-CH_3_	CH_3_	**4p**	80
17	5-Br	H	4-OCH_3_	CH_3_	**4q**	79
18	5-OCH_3_	H	4-Cl	CH_3_	**4r**	81
19	5-OCH_3_	H	4-Cl	H	**4s**	45

^a^Isolated yield.

^b^Yield for four consecutive cycles for recycled AgOTf were 86, 84, 80 and 78, respectively.

Then, we investigated the possibility of recycling of the catalyst. After the first cycle for model reaction, the solvent was concentrated under vacuum. Diethyl ether was added to the residue obtained and filtered leaving behind AgOTf. The recovered AgOTf was again taken in a round bottom flask and charged with 4-chlorobenzaldehyde (**1a**), *N*-methylaniline (**3**), and acetonitrile and allowed to react for 30 min followed by the addition of indole and reaction was allowed to continue for additional 90 min. The above sequence was repeated four times to give **4a **in good yields (88, 85, 83, and 80%) without much loss in catalytic activity of catalyst.

The reaction is assumed to proceed through two-step domino sequence. The first step is believed to be formation of iminium ion after reaction of the benzaldehyde and *N*-methylaniline. The next step is nucleophilic attack of indole on iminium ion followed by proton loss to give a 3-substituted indole. The structure of product is consistent with the synthesis of 3-aminoalkylated indoles *via *multicomponent condensation reaction of indoles, aldehyde, and amines [11-14].

### Anti-bacterial activity

An array of 20 diversely substituted indoles was evaluated for *in vitro *antibacterial activity against both Gram positive and Gram negative bacteria. The results of antibacterial activity of compounds (**4a-r**) are shown in Table 2. The compounds indicating notable antibacterial activity are indicated in bold (Table 2). Compounds **4q, 4r, 4i**, and **4b **showed significant antibacterial activity against Gram positive bacteria and **4r, 4b, 4o**, and **4l **against Gram negative bacteria. These results suggest that analog **4b **and **4r **can be used as potential broad spectrum antibacterial agents as they are potent against both Gram positive and Gram negative bacteria.

Compound **4d **(without functional groups) was not showing any antibacterial activity, however, substitution of electron withdrawing groups at phenyl ring (**4l, 4f**) exhibited increase in antibacterial activity against Gram negative organisms. Interestingly, introduction of electron-releasing group at phenyl ring (**4b**) showed good activity against both the Gram positive and Gram negative bacterial strains.

Among the compounds **4i-k**, the compound **4i **showed antibacterial activity against only *B. subtilis *but substituting the R'' position with an electron withdrawing group (**4j**, chloro) results in relatively less activity. In contrast, introducing an electron-releasing group at R'' position (**4k**, methoxy) made it further ineffective toward *B. subtilis *but found to be active against other two bacterial strains (Table 3).

**Table 3:** Zone of inhibition and MIC values of compounds against Gram positive and Gram negative bacteria

Compound	*E. coli*		*B. subtilis*		*S. aureus*	
	
	Zone of inhibition (mm)	MIC (μg ml^-1^)	Zone of inhibition (mm)	MIC (μg ml^-1^)	Zone of inhibition (mm)	MIC (μg ml^-1^)
**4a**	13	> 128	14	128	15	128
**4b**	16	**64**	16	**64**	10	> 128
**4c**	14	128	14	128	15	128
**4d**	13	> 128	14	128	13	128
**4e**	14	128	13	> 128	11	> 128
**4f**	15	128	12	> 128	10	> 128
**4g**	15	128	12	> 128	13	128
**4h**	13	> 128	14	128	11	> 128
**4i**	14	128	17	**64**	12	> 128
**4j**	13	128	15	128	12	> 128
**4k**	14	128	13	> 128	13	128
**4l**	15	**64**	12	> 128	14	128
**4m**	14	128	14	128	12	> 128
**4n**	14	128	14	128	13	128
**4o**	15	**64**	13	> 128	10	> 128
**4p**	13	128	14	128	12	> 128
**4q**	13	128	18	**64**	14	128
**4r**	16	**64**	17	**64**	14	128
Chloramphenicol	21	16	24	16	22	16

## Conclusion

In conclusion, we have developed an efficient and straightforward synthesis of 3-aminoalkylated indoles by one-pot three-component coupling reaction of a benzaldehyde, *N*-methylaniline, and indole or *N*-methylindole using AgOTf as catalyst. Simplicity, easy work up, short reaction time, environment friendly catalyst, and excellent yield are the advantages which will make this a practical method for synthesis of 3-aminoalkylated indoles over existing methods. All the synthesized compounds were evaluated for their antibacterial activities against both Gram negative and Gram positive bacteria. Compounds **4b **and **4r **showed good antibacterial activity against both Gram positive and Gram negative strains. However, inversing the property of substituent (from **4r **to **4q**) resulted in the significant fall in the magnitude of antibacterial activity against *E. coli*. This study provides insights for further optimizing of substituted indoles for the discovery of potent antibacterial agents.

## Competing interests

The authors declare that they have no competing interests.
